# Sustainable Strategies for the Agricultural Development of Shaanxi Province Based on the Risk Assessment of Heavy Metal Pollution

**DOI:** 10.3390/foods11101409

**Published:** 2022-05-13

**Authors:** Junhua Wu, Yiping Chen, Jifu Ma, Jing Cao, Yao Jiang

**Affiliations:** 1 State Key Laboratory of Loess and Quaternary Geology, Institute of Earth Environment, Chinese Academy of Sciences, Xi’an 710061, China; wujunhua@ieecas.cn (J.W.); caojing@ieecas.cn (J.C.); 2 University of Chinese Academy of Sciences, Beijing 100049, China; 3 College of Life Science, Yan’an University, Yan’an 716000, China; majf@ieecas.cn; 4 Xi’an Institute for Innovative Earth Environment Research, Xi’an 710061, China; viennay@163.com

**Keywords:** farmland soil, heavy metals, exposure risk assessment, pollution prevention and control, agricultural development

## Abstract

Heavy metal elements in farmland soil can be absorbed by crops and endanger food security. To assess the risk of heavy metal elements in farmland soil to crops in Shaanxi Province, we collected 693 soil samples and analyzed the concentrations of nine heavy metals (As, Hg, Pb, Cd, Cr, Mn, Cu, Zn, and Ni). According to the National Standard (GB 15619-2018) of the People’s Republic of China, the proportions of soil sample points in which the concentration of heavy metals was higher than the risk screening value were 2.02% (Cd), 0.29% (Cr), 0.29% (Zn), 2.31% (Cu), 1.15% (Ni), and 0.14% (Pb). The proportions of areas in which the concentration of heavy metal was higher than the background value were as follows, from largest to smallest: Zn (53.20%) > Mn (49.86%) > Cd (29.51%) > Hg (26.77%) > As (26.58%) > Ni (14.95%) > Cu (13.90%) > Pb (6.49%) > Cr (1.40%). The assessment of the risk of heavy metal exposure (geo-accumulation index (I_geo_), pollution load index (PLI), and potential ecological risk index (RI)) determined that Hg was the most concerning heavy metal in the farmland soil of Shaanxi Province. Moreover, 11.56% of these areas had Hg contamination, and they were mainly distributed in the western Guanzhong region. The farmland soil in the Guanzhong region was the most contaminated, followed by the southern Shaanxi region and then the northern Shaanxi region. The main sources of heavy metal contamination causing large-scale farmland soil pollution are agricultural production activities, transportation, and air pollution caused by coal combustion in Shaanxi Province. Therefore, sustainable strategies for the prevention and control of heavy metal pollution and agricultural development must be applied in different regions. Heavy metal pollution should be managed, and relevant policies should be created and enforced, such as the standardization of the use of qualified pesticides and fertilizers, improved treatment of livestock and poultry manure, development of the clean energy industry structure, and promotion of renewable energy vehicles. In terms of the high-quality development of agriculture, developing modern and local agriculture in different regions should be based on local geographical, climatic, and economic conditions.

## 1. Introduction

Food security is a serious issue involving not only the quantity of food but also the quality of food. In particular, the problems of food quality safety regarding levels of toxins in food are threatened by farmland soil contamination caused by pollutant discharge and pollutant migration worldwide. The exploitation of various mineral resources, metal smelting, agricultural activities, transportation, product manufacturing, waste water, waste gas, and solid waste discharge, as well as other human activities, has resulted in the discharge of significant amounts of toxic and harmful substances into the environment [1,2], and heavy metals are some of the primary pollutants. In 2007, 550 tons of As and 132 tons of Hg were emitted from coal-fired power plants in China. Every year, 5000 tons of Cu and 1200 tons of Zn are released into farmland as a result of fertilizer use [2,3,4]. In addition, heavy metals migrate and transform in the atmosphere, hydrosphere, and soil sphere, causing worldwide environmental pollution [5]. The rate of water pollution by heavy metals was as high as 80.1% in first decade of the 21st century in China [6,7]. Additionally, the concentration of heavy metals in soil far exceeded the background value in 21 cities [8,9]. The concentrations of As, Cr, Cu, Ni, and Pb in the soil surrounding a thermal power plant in Serbia were all higher than the standard value of soil, and the concentration of As was as high as 79.74 mg·kg^−1^ [10]. According to the “National Soil Pollution Survey Bulletin” of China published in 2014, the percentage of soil polluted by different heavy metals ranged from 0.9% to 7%, among which farmland soil was the most polluted. Heavy metals in soil are absorbed by plant roots and enter food chains, leading to serious health consequences [11,12,13,14]. According to Wong et al. (2002), between 10% and 20% of China’s farmland soil is contaminated with heavy metals, and pollution levels are increasing. Moreover, China is not the only country affected by soil contamination; many heavy metal concentrations are higher than the world average, e.g., in northern Bangladesh, the concentrations of Mn, Zn, As, and Pb were higher than the toxicity threshold in the agricultural soils [15]. Therefore, measures to ensure food quality safety are urgently needed across the globe.

Heavy metal elements accumulate in the human body and may lead to adverse health effects. For example, Cd exposure can cause liver injury, kidney failure, and bone softening deformity [16,17,18]. Exposure to Cr can cause allergic reactions on the skin, erosion of the skin (chromium sores), gastrointestinal ulcers, emphysema, and lung cancer [19,20,21]. The accumulation of Mn can cause neurological disorders, paralysis tremors, as well as severe or even permanent disability [22,23,24]. Pb poisoning can cause nervous system function disorder, cardiovascular damage, and renal cortical adenocarcinoma [25,26,27]. Exposure to Cu can cause acute gastroenteritis, renal failure, multiple neuritis, neurasthenia syndrome, and non-secretory pituitary tumors [28,29]. The accumulation of Zn can cause slow movement, anemia, diarrhea, arthritis, gastroenteritis, and other conditions [30,31]. Ni exposure can cause atopic dermatitis, pulmonary fibrosis, thymus atrophy, central circulation, and respiratory disorders [32,33,34,35]. As poisoning can inhibit cell oxidation and paralyze motor center function and often leads to skin cancer and peripheral vascular disease [36,37,38]. Hg poisoning can cause Minamata disease, teratogenic disorders, and immune system diseases [38,39,40].

Shaanxi Province is divided by the Beishan Mountain and Qinling Mountain into three distinct regions: northern Shaanxi, Guanzhong, and southern Shaanxi. These regions have different natural conditions, such as soil types, topography, and climate, with different patterns of agricultural production. Previous research on the heavy metal pollution in the farmland soil of Shaanxi Province has mainly focused on small representative areas [41,42,43,44]. Therefore, we analyzed the concentration and spatial distribution of heavy metals in farmland soil across the entire province and assessed the classification of risk exposure of heavy metals. In addition, the sources of heavy metal pollutants were identified, with the goal of providing sustainable strategies for agricultural development in Shaanxi Province.

## 2. Materials and Methods

### 2.1. Study Area and Sampling Site

Shaanxi Province has a mean annual precipitation of 649.4 mm. Its annual average temperature is 12.6 °C, and it is located between 105°29′–111°15′ E and 31°42′–39°35′ N. It has 397.7 × 10^4^ ha of cultivated land, 1116.4 × 10^4^ ha of woodland, and 286.8 × 10^4^ ha of grassland. There are ten prefecture-level cities and one demonstration area, with a total population of 39.55 million in Shaanxi Province. The northern Shaanxi region is located on the Loess Plateau, north of the Beishan Mountain. It is a region affected by the most serious soil erosion in the world [45]. The northern Shaanxi region, including the cities of Yan’an and Yulin, has an annual average temperature of 10 °C and annual precipitation of 614 mm. In terms of agricultural activity, the region had 107.2 × 10^4^ ha of land for major crops (e.g., grain crops, cotton, oil-bearing crops, fiber crops, sugar crops, flue-cured tobacco, and vegetables) and a total grain output of 336.05 × 10^4^ t in 2019. The Guanzhong region, including the cities of Tongchuan, Baoji, Xianyang, Xi’an, and Weinan, is located between the Beishan Mountain and Qinling Mountain. It has a warm temperate continental monsoon climate, with an annual average temperature of 14.3 °C and an annual precipitation of 493 mm. In 2019, it had 192.88 × 10^4^ ha of land for major crops and a total grain output of 689.58 × 10^4^ t. The southern Shaanxi region, including the cities of Hanzhong, Ankang, and Shangluo, is located south of the Qinling Mountain. It has an annual average temperature of 15.7 °C and an annual precipitation of 656 mm. In 2019, it had 113.31 × 10^4^ ha of land for major crops and a total grain output of 231.65 × 10^4^ t.

**Figure 1:**
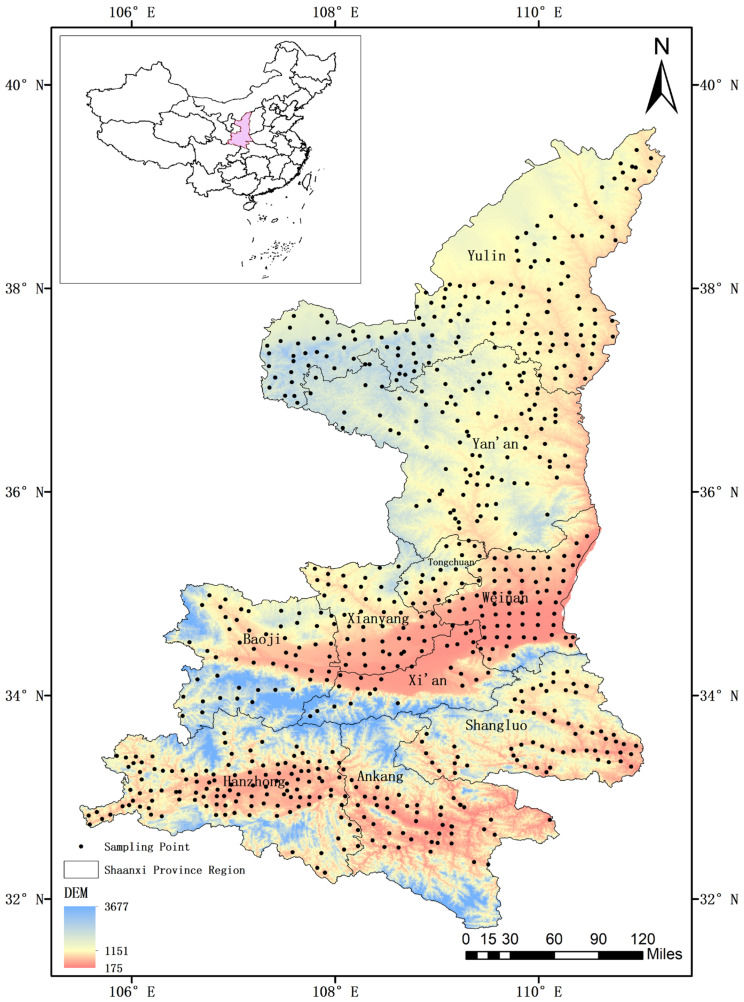
Distribution of soil sample collection points in Shaanxi Province.

For this study, farmland soil samples were collected by a sample point delimited method every 10 km, and no obvious source of point-source pollution (e.g., sewage outfalls and factories) was observed in the nearby area. Farmland soil (5 kg; 0–20 cm) from each of the four corners and the center of each quadrat (10 m × 10 m) of the sample point was mixed, of which 2 kg of soil was brought back to the laboratory and stored at 4 °C. A total of 693 farmland soil samples from Shaanxi Province were collected, as shown in Figure 1. In total, 208 samples from the Guanzhong region, 233 samples from southern Shaanxi, and 252 soil samples from northern Shaanxi were collected. The samples were dried at room temperature and then placed in an electric thermostatic drying oven (DHG-9070A, Shanghai Yiheng Scientific Instruments, China) to dry to a constant weight. After drying, the soil samples (50 g) were ground with an agate mortar and passed through a nylon sieve of 100 mesh (0.149 mm) prior to heavy metal analysis.

### 2.2. Sample Analysis

Soil samples (0.5 g) were accurately weighed and digested by standard mixed acid (HCl-HNO_3_-HF-HCLO_4_) using a conventional acid digestion method proposed in a previous study [46]. After digestion, 1 mL HNO_3_ and ultrapure water (18.2 MΩ/cm^2^ Milli-Q water, Millipore) were added and diluted to 50 mL. The acids used in the above processes were GR-grade.

The following atomic absorption spectroscopy techniques (AAS, ZEENit700P, Analytik Jena, Germany) were used to measure the concentration of heavy metals. The concentrations of Cu, Zn, Ni, Mn, and Cr were measured by flame atomic absorption spectroscopy. The concentrations of Pb and Cd were measured by graphite furnace atomic absorption spectroscopy, using a graphite furnace automatic sampler (Graphite Autosampler, MPE 60, Analytik Jena, Germany) and double magnetic field background correction. As and Hg were measured by hydride generation AAS (HS55, Analytik Jena, Germany).

The concentration of each sample was measured in triplicate, and the average value was calculated. The concentration of heavy metals in the samples was converted to mg per kg (mg·kg^−1^ dry weight). Blank and repeated samples were included simultaneously for each run. Thirty percent of the soil samples were randomly selected for repeated validation experiments, and the error range of the repeated validation experiments was kept within five percent. The limits of detection (LODs) were 0.011, 0.93, 0.41, 1.31, 2.03, 0.79, 0.095, 0.2, and 0.011 mg·kg^−1^ for Cd, Cu, Zn, Cr, Ni, Mn, Pb, As, and Hg, respectively. The limits of quantitation (LOQs) were 0.035, 2.8, 1.24, 3.93, 6.09, 2.36, 0.285, 0.6, and 0.034 mg·kg^−1^ for Cd, Cu, Zn, Cr, Ni, Mn, Pb, As, and Hg, respectively.

### 2.3. Risk Exposure Assessment of Heavy Metals

The classification of heavy metal concentrations in farmland soil was determined by the national standard: the Soil Environment Quality and Soil Pollution Risk Control Standard of Agricultural Land, GB 15619-2018 of the People’s Republic of China. Guanzhong and the southern Shaanxi and northern Shaanxi regions have different background values of heavy metals, which may affect the assessment of heavy metal exposure [47,48]. The risk assessment of heavy metal exposure was based on the geo-accumulation index (I_geo_), pollution load index (PLI), and potential ecological risk index (RI). The index was calculated as follows [49,50,51,52,53]:

(1) $$I_{\mathrm{geo}}=\log_{2}\left(\frac{C_{n}}{1.5\times B_{n}}\right)$$

The geo-accumulation index (Igeo) reflects the pollution status of single heavy metals in soil. C_n_ is the concentration of heavy metal elements; B_n_ is the background value of the heavy metal concentration in soil; and 1.5 is the correction coefficient. I_geo_ values in the range of <0, 0–1, 1–2, 2–3, 3–4, 4–5, and >5 refer to no pollution, mild pollution, mild to moderate pollution, moderate pollution, moderate to heavy pollution, heavy pollution, and severe pollution, respectively.

(2) $${\mathrm{CF}}_{i}=\frac{C_{i}}{B_{i}}$$

(3) $$\mathrm{PLI}=\sqrt[{n}]{{\mathrm{CF}}_{1}\times {\mathrm{CF}}_{2}\times {\mathrm{CF}}_{3}\times \ldots \times {\mathrm{CF}}_{n}}$$

The pollution load index (PLI) reflects the amount of pollution of nine heavy metals. C_i_ is the concentration of heavy metal element i, and B_i_ is the background value of heavy metal element i. The pollution load index is divided into four levels: PLI ≤ 1 indicates no pollution; 1 < PLI ≤ 2 indicates moderate pollution; 2 < PLI ≤ 3 indicates high pollution; and PLI ≥ 3 indicates extremely high pollution.

(4) $$C_{f}^{i}=C_{i}\;/{\;C}_{n}^{i}$$

(5) $$E_{r}^{i}=T_{r}^{i}\times C_{f}^{i}$$

(6) $$\mathrm{RI}=\sum E_{r}^{i}$$

The potential ecological risk index (RI) reflects not only the concentration of heavy metals but also their toxicity response coefficient linking the ecological, environmental, and toxicological effects of heavy metals together. $C_{f}^{i}$ is the single pollution index of heavy metal element i; C_i_ is the measured concentration of heavy metal element i; ${\;C}_{n}^{i}$ is the soil background value of heavy metal i; $E_{r}^{i}$ is the potential ecological risk index of heavy metal i; and $T_{r}^{i}$ is the toxicity response coefficient of heavy metal element i. The toxicity response coefficients of nine heavy metals were Mn = Zn = 1 < Cr = 2 < Cu = Ni = Pb = 5 < As = 10 < Cd = 30 < Hg = 40. The classification criteria were as follows: slight potential ecological risk (RI < 50), medium potential ecological risk (50 < RI < 100), relatively high potential ecological risk (100 < RI < 200), and high potential ecological risk (RI > 200).

### 2.4. Statistical Analysis

SPSS20.0 (IBM, Chicago, IL, USA) software was used for multivariate statistical analysis, including correlation analysis, principal component analysis, and cluster analysis, to provide a theoretical basis for the treatment of heavy metal pollution [54,55,56,57,58].

## 3. Results and Discussion

### 3.1. Spatial Distribution of Heavy Metals in Farmland Soil of Shaanxi Province

**Figure 2:**
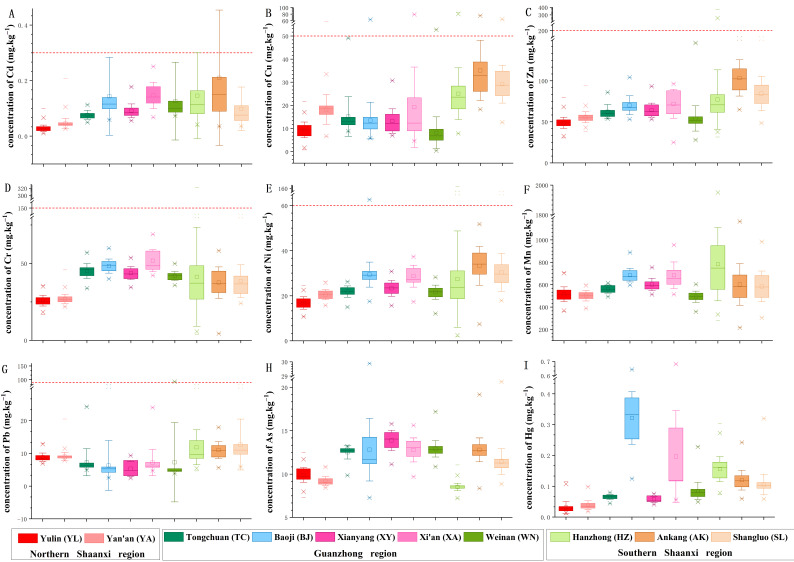
The concentrations (means ± standard deviation) of Cd (**A**), Cu (**B**), Zn (**C**), Cr (**D**), Ni (**E**), Mn (**F**), Pb (**G**), As (**H**), and Hg (**I**) in farmland soil of Shaanxi Province. The red lines indicate the risk screening value of the National Standard (GB 15618-2018) of the People’s Republic of China. The abbreviations are as follows: Yulin (YL, red), Yanan (YA, 50% red), Tongchuan (TC, dark cyan), Baoji (BJ, 50% dark cyan), Xianyang (XY, pink), Xi’an (XA, 50% pink), Weinan (WN, olive), Hanzhong (HZ, 50% olive), Ankang (AK, orange), and Shangluo (SL, 50% orange).

**Figure 3:**
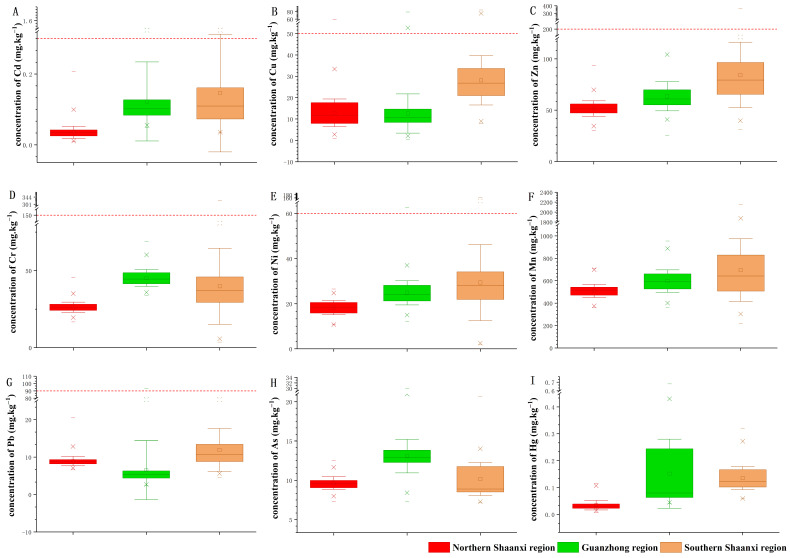
The concentrations (means ± standard deviation) of Cd (**A**), Cu (**B**), Zn (**C**), Cr (**D**), Ni (**E**), Mn (**F**), Pb (**G**), As (**H**), and Hg (**I**) in soil samples. The red lines indicate the risk screening concentration of the National Standard (GB 15618-2018) of the People’s Republic of China.

The concentrations (means ± standard deviation) of Cd, Cu, Zn, Cr, Ni, Mn, Pb, As, and Hg in the farmland soil of Shaanxi Province were 0.098 ± 0.123 mg·kg^−1^, 36.51 ± 16.80 mg·kg^−1^, 66.11 ± 24.77 mg·kg^−1^, 17.90 ± 11.75 mg·kg^−1^, 597.90 ± 190.70 mg·kg^−1^, 23.94 ± 11.34 mg·kg^−1^, 9.22 ± 5.89 mg·kg^−1^, 10.84 ± 2.29 mg·kg^−1^, and 0.10 ± 0.092 mg·kg^−1^, respectively (Figure 2). The concentrations of Cd, Zn, Ni, Mn, and Hg increased from the northern to the southern regions (Figure 3). The concentration of Cd in the northern Shaanxi region was the lowest (*p* < 0.05), while the concentrations of Cd in the Guanzhong and southern Shaanxi regions were relatively higher than that in the northern Shaanxi region, i.e., 0.035 ± 0.017 mg·kg^−1^, 0.119 ± 0.108 mg·kg^−1^, and 0.146 ± 0.166 mg·kg^−1^, respectively (Figure 3A). The Cu and Pb concentrations followed similar trends, where the concentration in the Guanzhong region was lower than that in both the southern and northern regions. However, for Cr and As, the concentrations in the Guanzhong region were higher than that of both the southern and northern regions (Figure 3D,H). Most concentrations did not exceed the risk screening values of the National Standard (GB 15618-2018 of the People’s Republic of China). The percentage of heavy metals with concentrations higher than the risk screening values (Figure 2) were 2.31% for Cu, 2.02% for Cd, 1.15% for Ni, 0.29% for Cr, 0.29% for Zn, and 0.14% for Pb, and these concentrations may pose a risk to crop growth, food quality, and even human health. This standard included the risk screening value for eight heavy metals except Mn. The risk screening values of As and Hg was much higher than the concentration of these two heavy metals in our research. Therefore, we did not show the risk screening value in Figure 2F,H,I. The percentage of sample points with Cu concentration higher than the risk screening value was 4.72%, 1.92%, and 0.40% in the southern Shaanxi, Guanzhong, and northern Shaanxi regions (Figure 2B), respectively. The sample points in which the Cd concentration was higher than the risk screening value were mainly distributed in the southern Shaanxi and Guanzhong regions, and the percentages were 4.72% and 1.44% (Figure 2A), respectively. In southern Shaanxi, 3.00% of samples had a Ni concentration higher than the risk screening value, and the Guanzhong region accounted for of 0.48% (Figure 2E). The concentrations of Cr and Zn exceeded the risk screening value only in the southern region, which accounted for 0.86% each (Figure 2C,D). The Pb concentration of only one sample was higher than the risk screening value in Weinan (Figure 2G). Based on the above results, there were six heavy metal (Cu, Cd, Ni, Cr, Zn, and Pb) concentrations that were higher than the risk screening values in Shaanxi Province. The proportion of heavy metal concentrations that were higher than the risk screening values was the highest in the southern region (Figure 2). The sampled points in which the concentrations were higher than the risk screening value were distributed in a way that suggested close proximity to point source pollution. Therefore, for those points, it is necessary to conduct field investigations to identify and mitigate the heavy metal pollution sources. In addition, ecological restoration of polluted farmland soil and perhaps alteration of the land use type are needed to ensure food safety [59,60,61].

**Figure 4:**
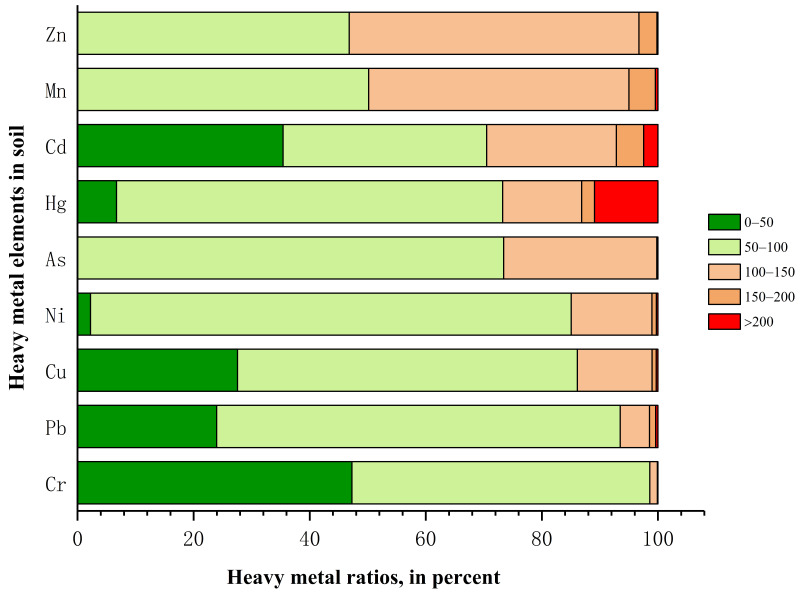
Percentage of soil samples with Zn, Mn, Cd, Hg, As, Ni, Cu, Pb, and Cr ratios (heavy metal element content/heavy metal background value ×100%) in Shaanxi Province. The classification standard of the ratios is as follows: level 1: the concentration is less than 50% of the background value (olive); level 2: the concentration is between 50% and 100% of the background value (50% olive); level 3: the concentration is between 100% and 150% of the background value (50% orange); level 4: the concentration is between 150% and 200% of the background value (orange); level 5: the concentration is greater than 200% of the background value (red).

The corresponding percentages of areas with heavy metal ratios (heavy metal element content/heavy metal background value ×100%) are shown in Figure 4. The percentages of areas (Figure 4) in which the concentration of heavy metals exceeded the background value are in the following order: Zn (53.20%) > Mn (49.86%) > Cd (29.51%) > Hg (26.77%) > As (26.58%) > Ni (14.95%) > Cu (13.90%) > Pb (6.49%) > Cr (1.40%). For these areas, Cd was mainly distributed in the southern Guanzhong region and the central part of southern Shaanxi. Cu was mainly distributed in the central and southwest parts of Yan’an, as well as the central and western parts of Xi’an and Ankang. Zn was mainly distributed in the southern part of the northern Shaanxi region, central and western Guanzhong, and most areas of the southern Shaanxi region. At higher concentrations than the background, Cr was found in only 1.40% of the farmland and was distributed in Xi’an and Hanzhong. Ni was mainly distributed to the west of Xi’an, the east of Baoji, the west of Hanzhong, and in central Ankang. Mn was mainly distributed in the central and eastern part of northern Shaanxi region, central and western Guanzhong, and most areas of the southern Shaanxi region. In total, 6.49% of samples contained Pb at concentrations higher than the background value, and Pb was mainly distributed near the Qinling Mountains and some areas of the southern Shaanxi region. As was mainly distributed in the central and northern areas of Guanzhong, as well as central and eastern southern Shaanxi. Hg was mainly distributed in the central part of the northern Shaanxi region, western and southern Guanzhong, and the western part of southern Shaanxi region. The percentage of areas with concentrations greater than two times the background value was (Figure 4): Hg (10.92%) > Cd (2.45%) > Mn (0.44%) > Pb (0.40%) > Cu (0.30%) > Ni (0.27%) > Zn (0.18%) > Cr (0.05%) > As (0.01%). The areas in which the concentration of Hg was higher than twice the background value were located in central and western Guanzhong, and these areas require close monitoring by the local authorities. Moreover, our results are consistent with previous research [62]. The distribution of farmland soil with concentrations of other heavy metals greater than twice the background value showed spot-like dispersion, mainly in the Guanzhong region and southern Shaanxi region.

### 3.2. Risk Exposure Index of Heavy Metals in Farmland Soil of Shaanxi Province

According to previous research of heavy metal pollution in a large region in Shaanxi Province, the Cd, Cr, Pb, Mn, and Zn concentrations all exceeded the standard to varying degrees. The percentage of Ni and Cu exceeding the standard was close to 100% in the soils of cultivated fruits and vegetables, as well as in nurseries, in the Chang’an district of Xi’an [63], because the sewage irrigation area of Xi’an accumulates heavy metals to different degrees [64]. In particular, Hg and Pb are considered serious pollutants in all points of the farmland soil in the suburbs of Xi’an. I_geo_ reached 1.37 and 1.23, respectively, and RI was as high as 501.25 [65]. While pollution by Cr, Ni, Pb, and P was found in the Guanzhong region, the overall pollution level was moderate [66].

**Figure 5:**
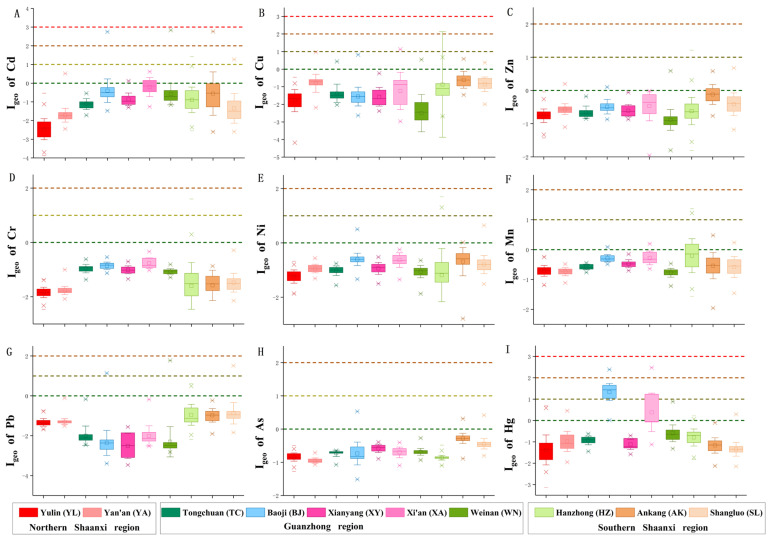
The geo-accumulation index (I_geo_) of Cd (**A**), Cu (**B**), Zn (**C**), Cr (**D**), Ni (**E**), Mn (**F**), Pb (**G**), As (**H**), and Hg (**I**) in soil samples. The pollution level standards of I_geo_ are Level 1 (I_geo_ < 0) pollution-free (green line); Level 2 (0 < I_geo_ < 1) mild pollution (yellow line); Level 3 (1 < I_geo_ < 2) mild-to-moderate pollution (orange line); Level 4 (2 < I_geo_ < 3) moderate pollution (red line). Abbreviations are as follows: Yulin (YL, red), Yanan (YA, 50% red), Tongchuan (TC, dark cyan), Baoji (BJ, 50% dark cyan), Xianyang (XY, pink), Xi’an (XA, 50% pink), Weinan (WN, olive), Hanzhong (HZ, 50% olive), Ankang (AK, orange), and Shangluo (SL, 50% orange).

**Figure 6:**
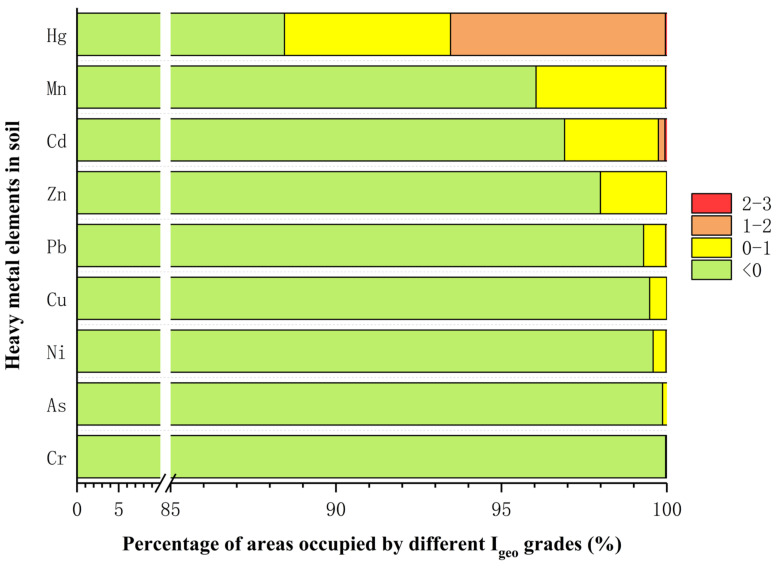
Geo-accumulation index (I_geo_) of Zn, Mn, Cd, Hg, As, Ni, Cu, Pb, and Cr in soil samples, in percent. The classification standards of I_geo_ are Level 1 (green) pollution-free (I_geo_ < 0); Level 2 (yellow) mild pollution (0 < I_geo_ < 1); Level 3 (orange) mild-to-moderate pollution (1 < I_geo_ < 2); and Level 4 (red) moderate pollution (2 < I_geo_ < 3).

**Figure 7:**
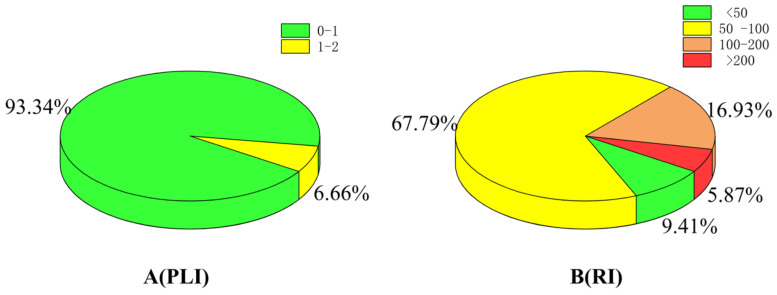
Percentage of areas with different grades of the soil pollution load index (**A**) and potential ecological risk index (**B**). The grading standards of PLI are as follows: Level 1 (green) pollution-free (PLI < 1); Level 2 (yellow) moderate pollution (1 < PLI < 2). The grading standards of RI are Level 1 (green) slight potential ecological risk (RI < 50); Level 2 (yellow) medium potential ecological risk (50 < RI < 100); Level 3 (orange) relatively high potential ecological risk (100 < RI < 200); and Level 4 (red) high potential ecological risk (RI > 200).

The geo-accumulation index (I_geo_), the pollution load index (PLI), and the potential ecological risk index (RI) of heavy metals were analyzed in farmland soil of Shaanxi Province. The spatial distribution pattern of the risk exposure indices (I_geo_, PLI, and RI) and percentage of farmland areas with different pollution levels were obtained (Figure 5, Figure 6 and Figure 7). In total, 11.56% of the tested areas in Shaanxi Province were contaminated with Hg (Figure 6), of which 5.02% were considered mildly polluted, 6.49% were mild to moderately polluted, and 0.05% were moderately polluted. These areas were mainly concentrated in the western Guanzhong region (Figure 5I). Of the areas tested, 3.96% were contaminated by Mn (Figure 6), of which 3.92% were mildly polluted and 0.04% were mild to moderately polluted. These areas were mainly distributed in the western part of southern Shaanxi and a few areas in the middle of the Guanzhong region (Figure 5F). In addition, 3.09% of the areas were contaminated by Cd (Figure 6), of which 2.83% were considered mildly polluted, 0.2% were mild to moderately polluted, and 0.06% were moderately polluted. These areas were distributed along either side of the Guanzhong region, as well as small areas in the center (west of Xi ‘an) of the region (Figure 5A). Moreover, the percentages of areas contaminated by Zn, Pb, Cu, Ni, As, and Cr were 2.01%, 0.70%, 0.52%, 0.41%, 0.13%, and 0.04%, respectively (Figure 6). According to the I_geo_, Hg contamination was the most concerning in farmland soil of Shaanxi Province, since 11.56% of areas had Hg contamination. These areas were mainly distributed in the western Guanzhong region (Figure 5I). Mn contamination was the second most serious threat to farmland soils (Figure 5F). Other heavy metals showed potential risk in some areas, and their distribution was dispersed, rather than clustered, throughout Shaanxi Province.

To the best of our knowledge, there have been very few large-scale pollution studies in Shaanxi Province; thus, our study is of great significance. However, our study lacks comparisons with previous studies. Through the statistical analysis of PLI, 93.34% of farmland soil was determined to be pollution-free (PLI < 1), and 6.66% of farmland soil was moderately polluted (1 < PLI < 2). These areas were mainly distributed in the central and western parts of the Guanzhong region (Xi’an and Baoji) and throughout the southern and northern Shaanxi regions. The results of potential ecological risk index (RI) hierarchical statistical analysis are shown in Figure 7B. From the analysis, 9.41% of farmland soils were classified as having a slight potential ecological risk (RI < 50), and these soils were mainly distributed in the northern part of Shaanxi Province (Yulin). In addition, 67.79% of farmland soils were classified as having a medium potential ecological risk (50 < RI < 100), and the distribution area for these soils was the most extensive. Moreover, 16.93% of farmland soils were classified as having a relatively high potential ecological risk (100 < RI < 200), and these soils were concentrated in the southern and western parts of Guanzhong region and scattered in northern and southern Shaanxi regions. Finally, 5.87% of farmland soils were classified as having a high potential ecological risk (RI > 200) and were mainly distributed in the northwest (Baoji) and central (Xi ‘an) parts of Guanzhong region.

Based on the PLI analysis, the farmland soil in the Guanzhong region poses the greatest risk to humans and the environment, due to heavy metal pollution, followed by the southern Shaanxi region and northern Shaanxi region. Guanzhong has the largest population, of 24.59 million, accounting for 63.44% of Shaanxi Province. It also has the largest economy, with a GDP of 1.61 trillion yuan, accounting for 63.20% of Shaanxi Province, and grain output of 6.9 million tons, accounting for 54.85% of Shaanxi Province (http://tjj.shaanxi.gov.cn/tjsj/ (accessed on 25 October 2021)). Compared with the northern and southern Shaanxi regions, Guanzhong has more human activities, and the environmental contamination cannot be ignored. Sustainable strategies should be implemented in the future.

### 3.3. Source Allocation of Heavy Metals in Farmland Soils of Shaanxi Province

**Figure 8:**
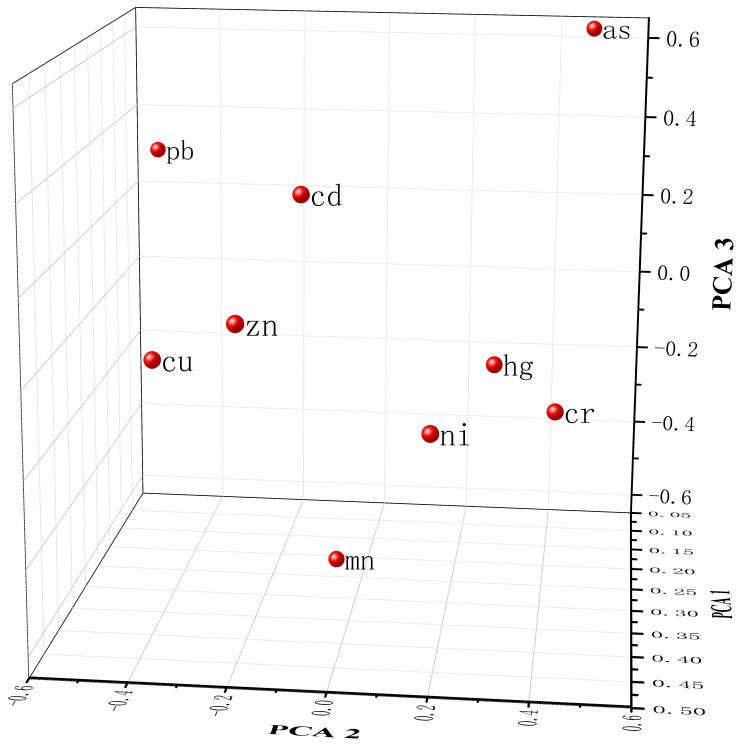
Principal component analysis (PCA) load diagram of heavy metal concentration in farmland soils of Shaanxi Province. There are three principal components greater than 1, and their characteristic values are 3.335, 1.414, and 1.038. Their cumulative variance contribution rates are 37.06%, 52.78%, and 64.31%.

**Figure 9:**
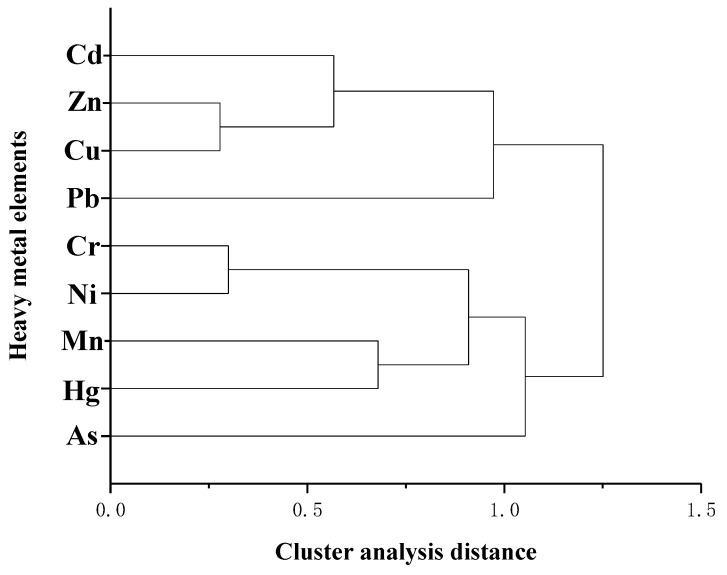
Cluster analysis of heavy metal concentration in farmland soils of Shaanxi Province.

The correlation analysis, principal component analysis, and cluster analysis of heavy metals in farmland soils of Shaanxi Province are shown in Table 1 and Table 2 and Figure 8 and Figure 9. The correlation of the spatial distribution of heavy metal concentration ranged from 0.519 to 0.732 (*p* < 0.01). The higher the correlation, the greater the possibility that the heavy metals came from of the same source. Cu–Zn, Hg–Cd, Ni–Zn, and Zn–Cd had the highest correlations, and Pb–Cu had the lowest correlation (Table 1). According to the load diagram of principal component analysis (Figure 8) and the total variance decomposition (Table 2), three principal components greater than 1 were obtained. The characteristic values of each component were 3.335, 1.414, and 1.038, and the cumulative variance contribution rates were 37.06%, 52.78%, and 64.31%. In terms of the first principal component, each heavy metal had different degrees of positive load values. The load values of Zn and Ni were larger at 0.46636 and 0.42939, respectively, while the load values of Pb and As were smaller at 0.11411 and 0.09501, respectively. In terms of the second principal component, the load values of Cr, Ni, As, and Hg were positive, while the load values of Cd, Zn, Cu, Mn, and Pb were negative. In terms of the third principal component, the load values of Cd, Zn, Pb, As, and Hg were positive, while the load values of Cr, Cu, Mn, and Ni were negative. By combining the above results with cluster analysis (Figure 9), heavy metals in farmland soils of Shaanxi Province were divided into three categories. The first category included Cd, Zn, Cu, and Pb. The second category included Cr, Ni, Mn, and Hg, and the third category consisted of As.

Parent rock material that weathers to form soil is a natural source of heavy metals that is closely related to geological and geomorphic properties and does not contribute to pollution. The three main sources of heavy metals in the farmland soil of Shaanxi Province are as follows: parent rock materials, agricultural activities and transportation, and air pollution from coal combustion. Agricultural activities and transportation are the main factors that lead to large-scale farmland pollution. Agricultural activities, such as pesticide and fertilizer use, livestock and poultry breeding, and sewage irrigation, result in the accumulation of Cu, Zn, Cd, and As in soils [67,68,69,70,71,72,73]. In terms of transportation processes, gasoline combustion, tire brake pad wear, and the loss of lubricating oil lead to Cd, Zn, and Pb contamination in the environment [74,75,76,77]. Although China has banned the use of leaded gasoline to reduce lead pollution, historical lead contamination must still be addressed [46]. Coal combustion produces Cr, Hg, As, Ni, Mn, and other heavy metal pollutants, which can be transmitted over long distances through atmospheric movement and enter the soil through atmospheric deposition [7,74,76,78,79,80]. Combined with the above-mentioned results of multivariate statistical analysis, the three categories of heavy metals were affected by the three pollution sources to varying degrees. The major sources of Cd, Zn, Cu, and Pb include parent rock material, agricultural activities, and transportation processes. The major source of Cr, Ni, Mn, and Hg is air pollution caused by coal combustion and parent rock materials. All three sources contributed to As contamination. Moreover, agricultural activities and transportation contributed the most, followed by coal combustion, and parent rock material contributed the least.

Based on the above results, sustainable strategies to prevent and control heavy metal contamination, as well as further agricultural development, must be applied in different regions based on local geographic, demographic, and economic conditions.

### 3.4. Sustainable Agricultural Development Pattern for Different Regions in Shaanxi Province

The three regions of Guanzhong, southern Shaanxi, and northern Shaanxi are threatened by heavy metal pollution from the above sources to different degrees, and one strategy to mitigate this contamination is to combine their geological and climate characteristics to promote high-quality agricultural development while ensuring food security.

According the ecological risk analysis, the establishment of control measures for pollution sources is necessary to ensure food safety. Therefore, we suggest the following: 1. Pesticides and fertilizers used must meet national safety and quality standards. In addition, the application amounts of chemical fertilizers and pesticides should be strictly controlled, as well as the safety of organic fertilizer produced by livestock and poultry manure. 2. The standardization of livestock and poultry breeding procedures should be reconsidered to implement feed and additives that meet national safety and quality standards, and livestock and poultry manure needs to undergo centralized treatment to render it harmless or be converted to organic fertilizer. 3. The use of new energy vehicles should be actively promoted. The exhaust emission standards of diesel vehicles in the entire region should be improved, and the progress toward more environmentally friendly agricultural production vehicles should be encouraged. 4. The standards for factory emissions and coal production should be raised. In addition, the development of clean energy industries, such as solar power generation and wind power generation, should be promoted to reduce the proportion of thermal power generation. Natural gas supply and central heating facilities should be built in rural areas to reduce the use of coal.

For the agricultural development of the Guanzhong region, its geographic, demographic, and economic advantages could be used to promote the application of advanced agricultural practices (i.e., automation, intelligent software, and real-time monitoring techniques) to prevent and control heavy metal pollution. The farmland soil of the Southern and northern Shaanxi regions had less heavy metal pollution, but the agriculture practices in these regions are limited by geographical and climatic factors, which restrict local agricultural development. The southern Shaanxi region has a suitable climate for the development of small-area and high-value agriculture that relies on modern technology, such as fungus cultivation by greenhouse technology and green tea cultivation under natural conditions. The northern Shaanxi region has been greatly affected by soil erosion and water loss, which also restrict the development of local agricultural. This area could benefit from the use of greenhouse technology, the development of fruit and vegetable industries, and agricultural production patterns suited for soil erosion areas, such as the ternary landscape pattern adopted in the eco-agricultural demonstration area of the Nangou village in Yan’an [81].

## 4. Conclusions

Through the calculation and analysis of heavy metals in farmland soil of Shaanxi Province, a risk assessment of heavy metal contamination as it pertains to food security and human health was established. Hg contamination was the most prevalent in farmland soils, and the farmland soil in the Guanzhong region was more threatened by heavy metal pollution than the other two areas. Agricultural activities, transportation, and air pollution were the sources of large-scale pollution in soils. Sustainable strategies for the prevention and control of heavy metal contamination, as well as the development of advanced agricultural practices, are urgently need in these three regions. Relevant policies should be formulated for pollution sources, such as the standardization of qualified pesticides and fertilizers, the development of environmentally friendly methods for livestock and poultry manure, and the promotion of a clean energy strategies. Agricultural development in these regions requires considering the combination of local geographical, climatic, and economic factors. For example, the Guanzhong region should develop high-tech agricultural practices; southern Shaanxi should develop high-value agricultural practices on the limited farmland; and northern Shaanxi should promote greenhouse technology in gentle sloping areas and institute production patterns suitable for areas with substantial soil erosion in hilly and gully areas. Based on our research, combined with the research on the physical and chemical nutrient properties of soils in Shaanxi Province, we can screen crops suitable for the local area, manage farmland more precisely, and promote the development of sustainable characteristic agriculture in Shaanxi Province.

## Figures and Tables

**Table 1:** Spearman correlation analysis of heavy metals in farmland soil of Shaanxi Province.

									*p* < 0.01
	Cd	Cr	Zn	Cu	Mn	Ni	Pb	As	Hg
Cd	1.000								
Cr	0.622	1.000							
Zn	0.678	0.454	1.000						
Cu	0.386	0.213	0.732	1.000					
Mn	0.415	0.414	0.536	0.350	1.000				
Ni	0.594	0.663	0.699	0.557	0.487	1.000			
Pb	−0.035	−0.217	0.258	0.519	0.001	0.069	1.000		
As	0.205	0.385	0.116	−0.201	0.035	0.256	−0.426	1.000	
Hg	0.724	0.546	0.574	0.370	0.435	0.555	0.009	0.078	1.000

**Table 2:** Load values of heavy metals from principal component analysis of Shaanxi Province soil samples.

	PCA1 (37.6%)	PCA2 (15.72%)	PCA3 (11.53%)
Cd	0.35901	−0.10052	0.35504
Cr	0.33914	0.4262	−0.1389
Zn	0.46636	−0.19254	0.13494
Cu	0.39838	−0.39258	−0.00376
Mn	0.2969	−0.04268	−0.55624
Ni	0.42939	0.18425	−0.12291
Pb	0.11411	−0.51865	0.31775
As	0.09501	0.47669	0.63714
Hg	0.29209	0.29164	0.0707

## Data Availability

The data presented in this study are available on request from the corresponding author. The data are not publicly available due to the manuscript contains a large amount of data, including some unpublished data.
